# Factors Influencing the Potential Distribution of Globally Endangered Egyptian Vulture Nesting Habitat in Nepal

**DOI:** 10.3390/ani13040633

**Published:** 2023-02-11

**Authors:** Hari Prasad Sharma, Santosh Dhakal, Krishna Prasad Bhusal, Hemanta Dhakal, Ramji Gautam, Ankit Bilash Joshi, Deu Bahadur Rana, Manshanta Ghimire, Suman Ghimire, Jerrold L. Belant

**Affiliations:** 1 Central Department of Zoology, Institute of Science and Technology, Tribhuvan University, Kirtipur, Kathmandu 44618, Nepal; 2 Bird Conservation Nepal, Kathmandu 44600, Nepal; 3 Department of Zoology, Prithvi Narayan Campus, Tribhuvan University, Pokhara 33700, Nepal; 4 Pokhara Bird Society, Pokhara, Kaski 33700, Nepal; 5 Department of National Parks and Wildlife Conservation, Babarmahal, Kathmandu 44600, Nepal; 6 Department of Fisheries and Wildlife, Michigan State University, East Lansing, MI 44824, USA

**Keywords:** foraging, modeling, *Neophron percnopterus*, roosting, suitable habitat, vulture

## Abstract

**Simple Summary:**

Potentially suitable habitat for nest-site selection of the globally endangered Egyptian vulture in Nepal was influenced primarily by precipitation and forested areas containing cliffs. Sites near to forests and human settlements were most suited habitat for Egyptian vultures in Nepal. Land-use change could negatively impact the nesting and distribution of Egyptian vultures and threaten their persistence in some areas. Potentially suitable nesting areas generally occurred outside of protected areas Therefore, to protect the potential nesting habitat of Egyptian vultures, forested areas with large numbers of cliffs should be conserved.

**Abstract:**

Habitat suitability is crucial to ensure the long-term persistence of species and can be identified based on relationships between species occurrences and underlying abiotic and biotic factors. We identified potential nesting habitat for the Egyptian vulture (*Neophron percnopterus*) in Nepal using ecological niche modeling with climatic variables. We estimated the currently suitable nesting habitat for Egyptian vulture in Nepal at 38,204 km^2^. We found a high probability of suitable nesting habitat on east-facing aspects, and the probability of a suitable nesting habitat was greater in more mountainous areas, particularly in central and western regions of Nepal. Precipitation was a major factor for predicting probability of the presence of nest sites for Egyptian vultures. After identifying potentially suitable habitat, we identified environmental factors affecting landscape-level suitable nesting habitat for Egyptian vultures using generalized linear models. For Egyptian vultures, sites near forests and human settlements were most suitable for nesting, roosting, and foraging, especially in central and western Nepal. Based on potentially suitable nesting habitat and previous work on Egyptian vulture foraging and roosting habitat, we recommend protecting forests near water sources and open areas for their long-term conservation.

## 1. Introduction

The Egyptian vulture (*Neophron percnopterus*) is a widely distributed species native to Asia, Africa, and Europe [1]. Egyptian vultures are categorized as endangered by the IUCN Red List of Threatened Species [1]. The estimated global population of Egyptian vultures is 12,400 to 36,000 individuals in the wild [1] and in Nepal; the species is non migratory, a widespread resident with an estimated population of 1000 individuals [2]. The global population of Egyptian vultures is decreasing rapidly (about 50–79% in three generations) including a 10% decline in Europe, 91% in Africa, 40–50% in Israel, and >40% in Greece in last three decades [1]. This species is already extinct from South Africa. Since 1999 this species has declined more than 35% annually in India [1,2]. Major threats include ingestion of the veterinary drug diclofenac, along with human disturbances, lead poisoning, electrocution, collisions, reduced food availability, and habitat change [1,3,4,5]. In Nepal, the Egyptian vulture has been assessed as vulnerable because of its population decline from diclofenac poisoning and other threats, including the destruction of nesting habitat, persecution, and other disturbances [2,6].

Vultures perform numerous beneficial activities including scavenging, which removes carcasses and can control the spread of disease [7]. However, the survival of many vulture species is threatened globally [1]. The causes of population decline vary geographically and include declines in carcass availability, habitat alteration, and poisoning [1]. In Nepal, vultures are currently threatened from forest degradation due to forest resource extraction [2], and disturbances to breeding, roosting, and feeding habitat [1,2]. Consequently, the government of Nepal developed a Vulture Conservation Action Plan in 2009 to prevent the potential loss of vultures, including Egyptian vultures, through the establishment of vulture safe-feeding sites and strengthening vulture-safe zones for providing diclofenac- and poison-free carcasses [8]. However, little information is available on suitable habitats for Egyptian vultures in Nepal, including nesting habitat.

Detailed information on the suitable habitat of Egyptian vultures is limited, and their nests are not uniformly distributed in Nepal [9]. Understanding the factors shaping species distributions and habitat suitability is critical to ensure their long-term persistence [10]. Species distribution models (SDMs) predict species distributions based on relationships between species occurrences and underlying abiotic and biotic factors [11,12]. Maps of species potential distributions generated from SDMs can contribute to conservation planning. For example, species potential distribution maps can help to identify habitat patches that may be connected to create functional meta-populations [11]. Identified suitable areas can also be used for the translocation of threatened species (e.g., the greater one-horned rhino (*Rhinoceros unicornis*)) [13,14] and to facilitate reserve designs (e.g., the important bird areas proposed for the Iberian Peninsula to conserve Golden Eagles (*Aquila chrysaetos*)) [15]. In addition, SDMs can be used to predict invasive species occurrences, nesting habitat, and threatened species distributions, the future prediction of species distribution based on climate change [16,17,18,19,20].

Nepal is a mountainous country containing many cliffs that provide a habitat for Egyptian vultures; therefore, we hypothesized that suitable nesting habitat for Egyptian vultures would be widely distributed throughout Nepal. We aimed to estimate the potential distribution of Egyptian vulture nesting habitat in Nepal. These baseline data can be used in support of Egyptian vulture management and conservation, particularly the biodiversity-conservation-related targets in the Vulture Conservation Action Plan (2015–2019), National Biodiversity Strategy and Action Plan of Nepal, and the National Parks and Wildlife Conservation Act 1973.

## 2. Materials and Methods

### 2.1. Study Area

**Figure 1:**
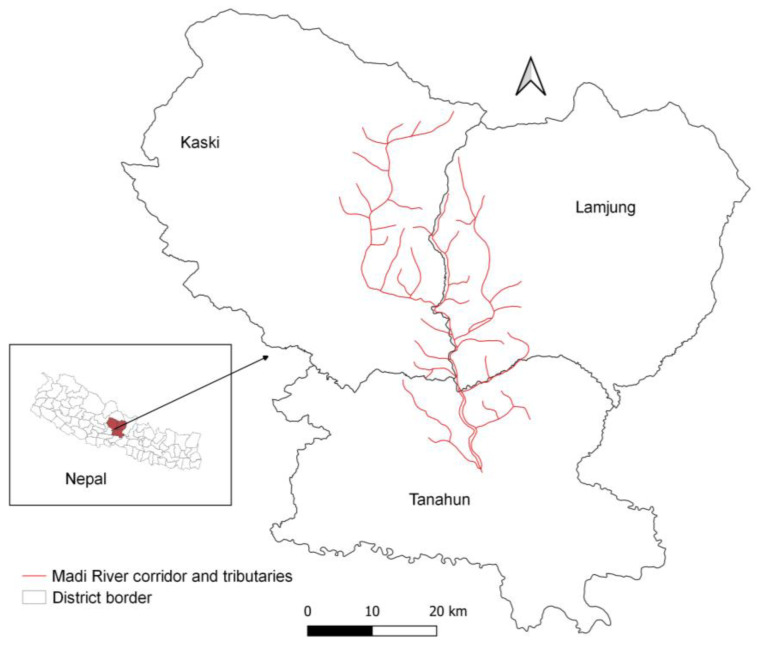
Madi River corridor and tributaries where field study was carried out after identifying potential suitable nesting habitat of Egyptian vultures in Tanahun, Kaski, and Lamjung districts, Nepal.

Nepal comprises 147,516 km^2^ (26°22′ to 30°27′ N and 80°04′ to 88°12′ E) and is bordered by China to the north and India to the south, east, and west. Elevations range from 60 to 8850 m above sea level. The climate varies among physiographic regions: the Tarai is considered tropical, the Siwalik subtropical, the Mid Hills and mountains are temperate, and the high mountain consists of subalpine and alpine climates [21]. Varied climatic zones of Nepal support the occurrence of more than 17,097 faunal species, including 886 bird species, of which nine are vulture species [22]. The Madi River corridor occurs in Tanahun, Kaski, and Lumjung districts in the Gandaki Province of Nepal (Figure 1) between the Tarai and High Mountains at an elevation range of 300–8155 m above the sea level. This corridor contains many steppe areas; dominant tree species include needlewood (*Schima wallichii*), sal (*Shorea robusta*), chestnut (*Castanopsis indica*), pine (*Pinus wallichiana*), banyan (*Ficus benghalensis*), pipal tree (*Ficus religiosa*), red cedar (*Toona ciliate*), gular (*Ficus racemosa*), red silk cotton (*Bombax ceiba*), and Indian rosewood (*Dalbergia sissoo*). In addition to Egyptian vultures, wildlife occurring in the Madi River corridor includes leopards (*Panthera pardus*), Himalayan black bears (*Ursus thibrtanus*), Assamese monkeys (*Macaca assamensis*), golden jackals (*Canis aurens*), jungle cats (*Felis chaus*), yellow-throated martens (*Martes flavigula*), and crab-eating mongooses (*Herpestes urva*) [23,24]. Overall, 992,110 people inhabit the Tanahun, Kaski, and Lumjung districts of Nepal [25].

### 2.2. Study Species

The Egyptian vulture is an old-world vulture and a long-lived scavenger. The body measures 47–65 cm from beak tip to tail feathers, with a wingspan about 2.7 times longer than its body length [26]. Adult plumage is white with black flight feathers on the wings and yellow faces; however, sub-adults possess black plumage distinct from adults and other birds. The beak is slender and hook-shaped, adapted for removing meat from carcasses. Males are slightly smaller than females, with body mass ranging from 2.0 to 2.5 kg [27]. Egyptian vulture diets are diverse and include carrion, tortoises, organic waste, insects, young vertebrates, eggs, and feces [28]. Egyptian vultures are solitary nesters and monogamous, with a breeding season from February through May. They generally use the same nest each year, constructed on cliffs and rocky outcrops, but occasionally nest in large trees and utility poles [29,30].

### 2.3. Data Collection and Management

We obtained 60 locations of active nests of Egyptian vultures throughout Nepal 2006–2021. The nearest distance between nests was 3 km. We obtained nest location data mainly during the breeding season (late February–May). Breeding location of the Egyptian Vultures was checked during field surveys 2020–2021. We used a nest with either eggs or nestlings as an indicator of confirmed breeding. These data were collected during regular vulture-nest monitoring conducted by biologists of the Bird Conservation Nepal, Pokhara Bird Society and supplemented with other research on this species (e.g., [6,31,32] and under the project of Division Forest Kaski, Gandaki Province, Nepal.

### 2.4. Spatial Modeling

We used species distribution models which envisage potentially suitable habitat for species based on variables supportive to species occurrences [33]. Numerous model types are available for developing SDMs; however, maximum entropy (e.g., Maxent) is one of most effective tools for species having limited spatial presence-only data [34,35,36]. Maximum entropy forecasts the species probability distribution of random events with uniform and high stability [37,38].

**Figure 2:**
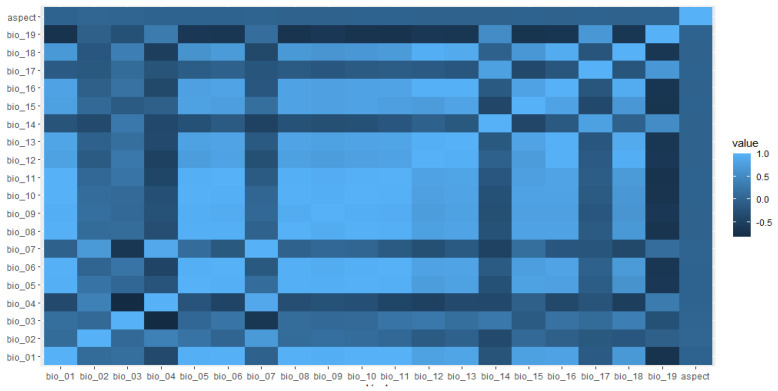
Spearman pairwise correlation coefficients between predictive variables aspect, mean annual temperature (bio_01), mean diurnal range (bio_02), iso-thermality (bio_03), temperature seasonality (bio_04), maximum temperature of the warmest month (bio_05), minimum temperature of coldest month (bio_06), temperature annual range (bio_07), mean temperature of wettest quarter (bio_08), mean temperature of driest quarter (bio_09), mean temperature of the warmest quarter (bio_10), mean temperature of coldest quarter (bio_11), annual precipitation (bio_12), precipitation of wettest month (bio_13), precipitation of driest month (bio_14), precipitation seasonality (bio_15), precipitation of driest quarter (bio_16), precipitation of wettest quarter (bio_17), precipitation of the warmest quarter (bio_18) and precipitation of the coldest quarter (bio_19) considered in the Egyptian vulture nesting habitat distribution model, Nepal. Color variation indicates correlation values.

We determined potential habitat layers by first selecting 19 bioclimatic variables at 30 arc-second (about 1 km^2^) resolution from Worldclim [39] and extracted aspect from a digital elevation model (about 1-km^2^ resolution; Shuttle Radar Topography Mission (SRTM); https://ita.cr.usgs.gov, accessed on 28 November 2022) (Supplementary Table S1). We selected eight environmental variables including mean annual temperature (Bio_1), mean diurnal range (Bio_02), iso-thermality (Bio_3), annual precipitation (Bio_12), precipitation of driest month (Bio_14), precipitation seasonality (Bio_15), precipitation of coldest quarter (Bio_19), and aspect (see Figure 2) based on jackknife analysis, which uses a leave-one-out approach to estimate variable importance (Supplementary Materials Table S1; Figure S1). We excluded highly correlated (|r| > 0.70) variables to reduce the potential for model overfitting [40]. We used maximum entropy modeling software (Maxent version 3.3.3 K) [36] to estimate the potential nesting distribution of Egyptian vultures.

We ran Maxent models using 75% presence data for calibration and the remaining 25% for model validation [41] using the bootstrapping procedure with 10 replications and a maximum of 500 iterations. We used the default settings for the number of background samples and auto features for model construction. We evaluated model validation and accuracy using the area under the curve (AUC) of the receiver operating characteristics [42]. However, because of criticism in the use of AUC for model evaluation [43], we also evaluated model performance using the true skill statistic (TSS), sensitivity, and specificity [44]. The TSS values range from −1 to 1, with values approaching 1 indicating good model performance and values <0 indicating performance no better than random. We converted habitat into binary form (suitable or unsuitable) using the 10th-percentile training presence logistic threshold [45] and used the corresponding raster layer to identify suitable potential nesting habitat for Egyptian vultures.

We used predicted suitable nesting habitat to identify whether the model correctly predicted nesting habitat as well as existing factors influencing the occurrence of Egyptian vulture nests along the Madi River corridor from Damauli, Tanahu to Tanting, Kaski. This area supports abundant Egyptian vultures and we assumed this abundance reflected their residence in this area, where cliffs and forests occur near human settlements. We established 35 stations at 3-km intervals along 135 km of the Madi River. Each station was 500 m × 500 m and within each we established five 100 m × 100 m plots, four at the corners and one at the center. During October 2020–February 2021, we searched each plot for 3 h, recording the occurrence of Egyptian vulture nests and the presence of Egyptian vultures roosting or feeding. We recorded the occurrence of feeding vultures while sitting quietly at the corner of each plot, and for observations of nesting and roosting we searched throughout the plots. From the center of each plot we recorded the elevation using GPS, and measured the distance to the nearest forest, area of agricultural land, water, and human settlement. We measured the nearest distance to human settlement using a GIS if the area had more than one house and were used for living.

### 2.5. Data Analysis

We used generalized linear models to estimate the effects of distance to forest, distance to agricultural land, distance to nearest water, and distance to human settlement on the presence of nesting and feeding/roosting vultures. We ranked models using the Akaike information criterion adjusted for small samples [46] and Akaike model weights to estimate the relative strength of evidence for each model. We considered models with AICc scores within 4 of the most parsimonious model to have support [46]. We conducted model averaging using all models within 4 AICc of the top model to estimate 95% confidence intervals for each variable and accepted statistical significance at α = 0.05. All analyses were performed in the program R [47].

## 3. Results

**Figure 3:**
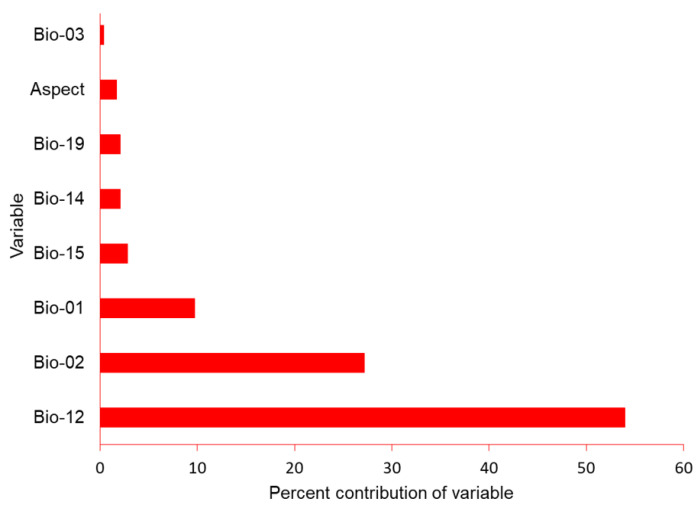
Percent contribution of climactic variables aspect, mean annual temperature (bio_01), mean diurnal range (bio_02), iso-thermality (bio_03), annual precipitation (bio_12), precipitation of wettest month (bio_13), precipitation of driest month (bio_14), precipitation seasonality (bio_15) and precipitation of the coldest quarter (bio_19) for model construction for the potential distribution of suitable nest habitat for Egyptian vultures, Nepal.

We obtained the locations of 60 nest sites for use in the SDM. Maxent had good performance as indicated by the high AUC (0.952), TSS (0.600), sensitivity (0.869), and specificity (0.730). Annual precipitation (Bio_12) contributed 54%, followed by mean diurnal range (Bio_02: 27%), and mean annual temperature (Bio_1: 8%) (Figure 3).

**Figure 4:**
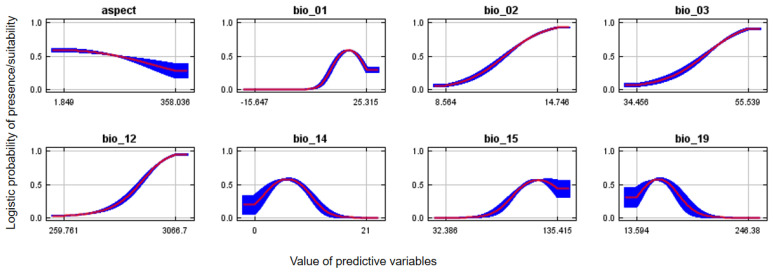
Response curves of aspect, mean annual temperature (bio_1), mean diurnal range (bio_02), iso-thermality (bio_3), annual precipitation (bio_12), precipitation of driest month (bio_14), precipitation seasonality (bio_15) and precipitation of coldest quarter (bio_19) on the probability of potential suitable nest habitat for Egyptian vultures, Nepal.

**Figure 5:**
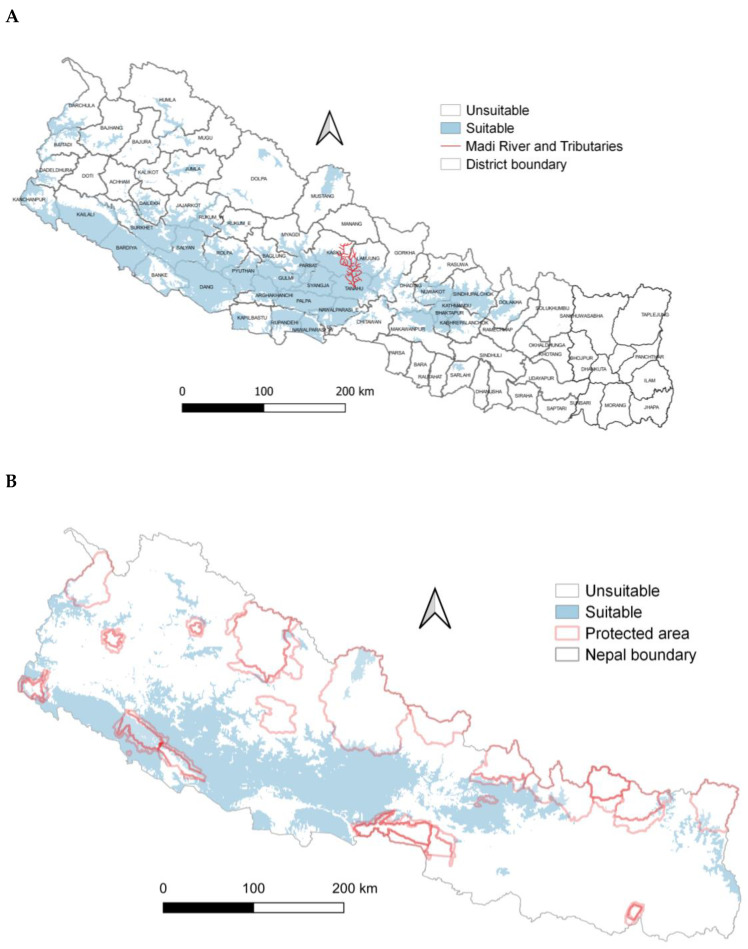
(**A**) Potential suitable nest habitat of Egyptian vultures, Nepal. (**B**) Potential suitable nest habitat of Egyptian vultures and protected areas, Nepal.

The greatest probability of Egyptian vulture potential nest habitat suitability for annual precipitation was within the range of 268–3008 mm and annual temperatures of 20–33 °C. (Figure 4). The probability of suitable nesting habitat was greater on east-facing aspects. The probability of suitable Egyptian vulture nesting habitat was greater in central and western regions than other parts of the country and in more mountainous areas, and overall comprised 38,204 km^2^ (Figure 5A). Suitable areas mostly occurred outside protected areas (Figure 5B).

Among 175 sampling plots in the predicted suitable habitat, Egyptian vultures were sighted at 56 plots (32%). We observed Egyptian vultures feeding (*n* = 17), soaring (*n* = 33), and roosting (*n* = 36), but no nests were detected. The occurrence of Egyptian vultures in their natural habitat was greater near forests (Table 1 and Table 2). The best model included areas near forests and water. Proximity to human settlement also positively influenced the occurrence of Egyptian vultures. The probability of Egyptian vultures’ presence increased with increasing distances to agricultural land and slightly with increasing distance to water. 

## 4. Discussion

The Egyptian vulture’s nesting distribution appears to be influenced by climatic, environmental, and anthropogenic factors. Among climatic factors, annual precipitation contributed the most, which could be a result of abundant precipitation delaying rapid carcass depletion that benefits vulture scavenging [48]. The nests of Egyptian vultures are more common in the central and western mid-hill regions [49,50,51] and high annual precipitation occurs these areas [52]. In these areas, rubbish dumps are common in most municipalities, which undoubtedly attract Egyptian vultures for feeding (Sharma, H.P. personal observation). East-facing slopes also were more likely to have Egyptian vulture nest sites, and increased temperatures from earlier sun exposure could promote thermals to aid flight earlier in the day to facilitate foraging [53].

We were unable to detect nests while searching in the predicted suitable nest area; however, we were able to find foraging and roosting Egyptian vultures in the study area. The majority of suitable habitats were located in the central and western regions, probably due to vulture safe-feeding sites in these regions, which provide diclofenac- and poison-free food to the vultures [8]. The distribution and population of Egyptian vultures inside the protected areas of Nepal is little known. The majority of the protected areas seem less supportive for vulture conservation, so that the government of Nepal declared a vulture safe-feeding zone throughout Nepal for vulture conservation [8]. We found Egyptian vultures selected nest sites close to the forest, likely in part to facilitate nest construction. The Madi River corridor is in the Mid Hills region with a steppe habitat, which might provide visibility for foraging [54]. There is a greater prevalence of steep cliffs in forests of the Mid Hills region (e.g., Kali Gandaki and Madi river corridors) than other regions of Nepal, where vultures construct nests on cliffs and in nearby trees [55,56]. Cliffs can be advantageous to Egyptian vultures due to the greater survival of young in cliff nests and the greater visibility of carrion at lower elevations (Bhusal, K. personal observation). The nesting and roosting of Egyptian vultures on cliffs and trees high above the ground has been previously reported [57]. In addition, a higher probability of the presence of nests near the forest edge could be due to greater diversity and availability of food, including small mammals, young birds, fish, eggs, and decomposed fruits, all of which are consumed by Egyptian vultures [58]. The probability of Egyptian vultures’ nest habitat was not influenced by the distance to water. Forests and cliffs are more prevalent near water, especially rivers (Sharma, H. P., personal observation), and water may be important for vultures to clean the waste from feeding on carcasses and human refuse [59,60]. It is possible that the unimportance of water was an artifact of our study area occurring within a river corridor in which water was not limited.

The distance to agricultural land did not influence the occurrence of Egyptian vultures. In Nepal, people currently bury dead livestock; therefore, carcasses are not available in agricultural land in comparison to forests. That Egyptian vultures were more likely to be present near human settlements is also likely due to greater food availability, including slaughterhouses and dumping sites near settlements in the Mid Hills region [6]. However, human disturbances including roads and associated vehicle traffic can reduce vulture occurrence [49,61].

We acknowledge several limitations in our study. First, we were uncertain whether we detected every vulture or nest that was present or whether multiple observations represented the same individual vulture. However, that we detected multiple nests used in our initial model but none during our assessment for model validation suggests that few, if any, nests were undetected. Further, that an individual bird was observed in multiple plots would not influence our results, as we were addressing population-level responses to various factors that influence vulture presence. Finally, we note that our use of aspect as a continuous variable may have been less desirable than use of alternatives (e.g., cardinal directions), and may require a larger sample size than that which we used.

We developed the first national map for Nepal that estimates areas of suitable nesting habitat for Egyptian vultures. This research identifies areas of potential nesting sites and identifies factors influencing the presence of the species. Our results support the post-1990 distribution of Egyptian vultures, which was estimated to be most limited in east-central and eastern Nepal [2]. Our study also further informs and supports the provisional vulture safe zone, a pioneering landscape conservation approach for vultures in Nepal [62].

## 5. Conclusions

The nest-site selection of Egyptian vultures was influenced by nearest forests for nest construction and also likely near to human settlement. Based on these factors acting on the occurrence of Egyptian vultures, we have provided the first estimate of its potential persistent nesting distribution in Nepal. Climate, but more likely land-use change, could limit nesting habitat and the overall distribution of this species and threaten its persistence in some areas. Importantly, potential suitable nesting areas occurred predominantly outside of protected areas and we recommend further safeguards to protect the nesting habitat of Egyptian vultures, particularly maintaining forested areas with nearby cliffs, a recommendation within the Vulture Conservation Action Plan of Nepal (2015–2019).

## Figures and Tables

**Table 1:** Outputs from generalized linear models to identify factors related to the probability of presence of Egyptian vultures, Madi River corridor, Nepal. Egyptian vulture (presence) used as response variable; elevation (m); distance to forest (m), distance to agricultural land (m), distance to human settlement (m), distance to water (m) are predictive variables. K is the number of parameters, ∆AICc is the difference between the AICc value of the best-supported model and successive models, and wi is the Akaike model weight. Only models with ∆AICc < 4 are reported.

Parameters	K	logLik	AICc	∆AICc	w_i_
Distance to forest + Distance to water	3	−37.08	80.20	0.00	0.12
Distance to forest + Distance to water + Distance to settlement	4	−36.26	80.50	0.36	0.10
Distance to forest + Distance to water + Distance to settlement + Elevation	5	−35.32	80.60	0.46	0.09
Distance to forest + Distance to water + Elevation	4	−36.52	81.00	0.87	0.08
Distance to agricultural land + Distance to forest + Distance to water + Distance to settlement	5	−35.97	81.90	1.76	0.05
Distance to forest + Elevation	3	−37.98	82.00	1.79	0.05
Distance to water	2	−39.03	82.00	1.86	0.05
Distance to agricultural land + Distance to forest + Distance to water	4	−37.07	82.10	1.97	0.04
Distance to forest + Distance to settlement + Elevation	4	−37.17	82.30	2.18	0.04
Distance to agricultural land + Distance to forest + Distance to water + Distance to settlement + Elevation	6	−35.26	82.50	2.36	0.04
Distance to water + Elevation	3	−38.34	82.70	2.51	0.03
Distance to agricultural land + Distance to forest + Distance to water + Elevation	5	−36.36	82.70	2.54	0.03
Distance to water + Distance to settlement + Elevation	4	−37.48	83.00	2.78	0.03
Distance to water + Distance to settlement	3	−38.51	83.00	2.85	0.03
Distance to forest	2	−39.67	83.30	3.16	0.02
Distance to agricultural land + Distance to forest + Elevation	4	−37.73	83.50	3.29	0.02
Elevation	2	−39.78	83.60	3.39	0.02
Distance to agricultural land + Distance to water	3	−39.01	84.00	3.84	0.02
Distance to agricultural land + Distance to water + Distance to settlement	4	−38.03	84.10	3.89	0.02

**Table 2:** Model-averaged parameter estimates and lower and upper 95% confidence limits describing the occurrence of Egyptian vultures in Nepal. Parameter estimates were averaged from all models reported in Table 1.

Variables	Estimate	SE	Lower CL	Upper CL	z	*p*
(Intercept)	1.367	0.794	−0.190	2.10086	1.721	0.085
Distance to forest	−0.005	0.002	−0.009	0.00004	1.976	0.048
Distance to water	0.004	0.003	−0.001	0.0101	1.533	0.125
Distance to settlement	−0.002	0.002	−0.005	0.0010	1.295	0.195
Elevation	0.001	0.001	−0.001	0.0029	1.344	0.179
Distance to agricultural land	0.0003	0.002	−0.004	0.0049	0.118	0.906

## Data Availability

Not applicable.

## Supplementary Materials

The following supporting information can be downloaded at: https://www.mdpi.com/article/10.3390/ani13040633/s1, Figure S1: Jackknife analysis used to select predictive climactic variables based on their importance to model potential suitable nest habitat of Egyptian vultures, Nepal; Table S1: Predictive variables considered in model to estimate potential distribution of suitable nest habitat for Egyptian vultures, Nepal.
