# Supplementary material for: Factors Influencing the Potential Distribution of Globally Endangered Egyptian Vulture Nesting Habitat in Nepal
Source: Animals (Basel). 2023 Feb 11;13(4):633. doi: 10.3390/ani13040633 (PMC9951647; doi:10.3390/ani13040633)
Supplement: Supplementary file 1 [file animals-13-00633-s001.zip › animals-2092897-supplementary.pdf]

Table S1. Predictive variables considered in model to estimate potential distribution of suitable nest habitat for Egyptian vultures, Nepal. The bold variables were included in the model after correlation analysis (|r| < 0.7).

Variable Code	Variable
**BIO_1**	**Annual mean temperature**
**BIO_2**	**Mean diurnal range (monthly mean, T° max-T° min)**
**BIO_3**	**Iso-thermality (bio2/bio7) x 100**
BIO_4	Temperature seasonality (standard deviation × 100)
BIO_5	Maximum temperature of the warmest month
BIO_6	Minimum temperature of coldest month
BIO_7	Temperature annual range (bio5-bio6)
BIO_8	Mean temperature of wettest quarter
BIO_9	Mean temperature of driest quarter
BIO_10	Mean temperature of the warmest quarter
BIO_11	Mean temperature of coldest quarter
**BIO_12**	**Annual precipitation**
BIO_13	Precipitation of wettest month
**BIO_14**	**Precipitation of driest month**
**BIO_15**	**Precipitation seasonality (coefficient of variation)**
BIO_16	Precipitation of wettest quarter
BIO_17	Precipitation of driest quarter
BIO_18	Precipitation of the warmest quarter
**BIO_19**	**Precipitation of the coldest quarter**
**Aspect**	**Continuous**

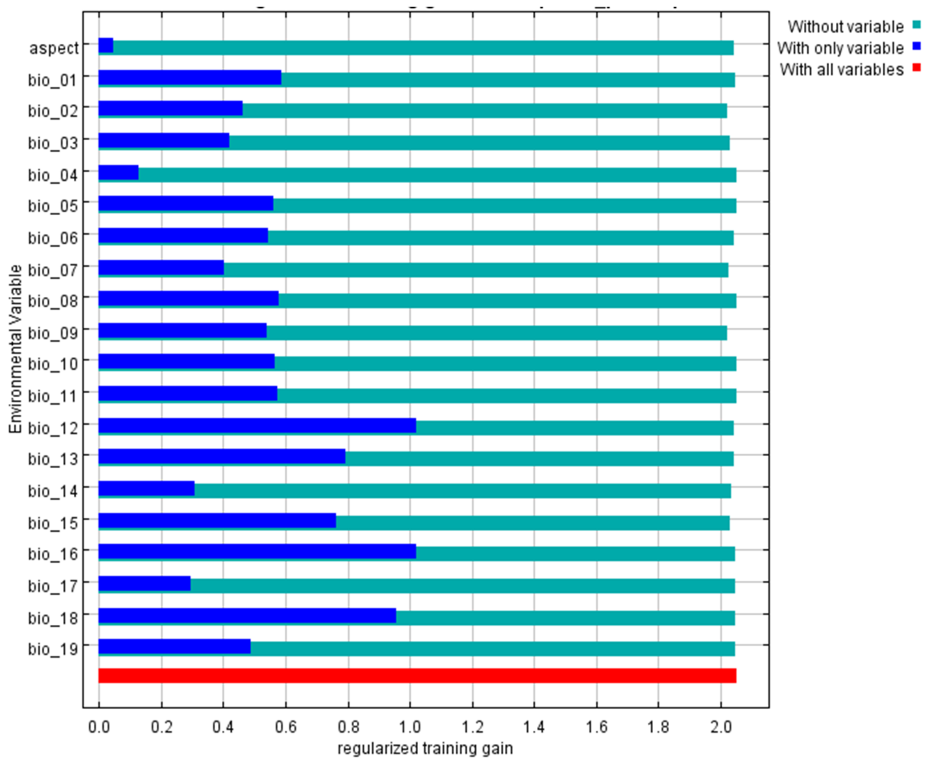

Figure S1. Jackknife analysis used to select predictive climactic variables based on their importance to model potential suitable nest habitat of Egyptian vultures, Nepal. See Supplementary Table S1 for variable descriptions.
